# The Multifaceted Complexity of Tumor Necrosis Factor Receptor-Associated Periodic Syndrome (TRAPS): A Case Report Highlighting Atypical Gastrointestinal Manifestations

**DOI:** 10.3390/diagnostics14131337

**Published:** 2024-06-24

**Authors:** Massimiliano Mancini, Giovanni Di Nardo, Emanuele Casciani, Maria Letizia Feudi, Lavinia Bargiacchi, Angelica Petraroli, Francesca Della Casa, Arianna Di Napoli, Andrea Vecchione

**Affiliations:** 1 Morphologic and Molecular Pathology Unit, Sant’Andrea University Hospital, 00189 Rome, Italy; mamancini@ospedalesantandrea.it (M.M.); mfeudi@ospedalesantandrea.it (M.L.F.); 2 Department of Neurosciences, Mental Health and Sensory Organs (NESMOS), Faculty of Medicine and Psychology, Sapienza University of Rome, Pediatric Unit, Sant’Andrea University Hospital, 00189 Rome, Italy; giovanni.dinardo@uniroma1.it; 3 University of Rome Tor Vergata—Casa di Cura Pio XI, 00133 Rome, Italy; emanuelecasciani@gmail.com; 4 Department of Medical and Surgical Sciences and Translational Medicine, Faculty of Medicine and Psychology, Sapienza University of Rome, 00189 Rome, Italy; lavinia.bargiacchi@uniroma1.it; 5 Department of Translational Medical Sciences, University of Naples Federico II, 80133 Naples, Italy; ambulatoriopetraroli@gmail.com (A.P.); francesca.dellacasa4@gmail.com (F.D.C.); 6 Department of Clinical and Molecular Medicine, Sant’Andrea Hospital, 00189 Rome, Italy; arianna.dinapoli@uniroma1.it

**Keywords:** TRAPS, autoinflammatory disease, intestinal obstruction, amyloidosis

## Abstract

Background. Tumor Necrosis Factor Receptor-Associated Periodic Syndrome (TRAPS) is an autosomal dominant autoinflammatory disorder stemming from mutations in the TNFRSF1A gene affecting the tumor necrosis factor receptor (TNFR)-1. These mutations lead to dysregulated inflammatory responses, primarily mediated by augmented interleukin (IL)-1β release. Case Presentation. We present the case of a 29-year-old woman with a history of recurrent febrile episodes, abdominal pain, and joint manifestations, eventually diagnosed with TRAPS following genetic testing revealing a heterozygous R92Q mutation in TNFRSF1A. Further genetic examinations unveiled additional clinically significant mutations, complicating the clinical picture. Our patient exhibited delayed colonic transit time and right colonic amyloidosis, a rare complication. Surgical intervention was required for overwhelming intestinal obstruction, revealing mucosal atrophy and dense lymphocytic infiltrates on histological examination. Discussion. Gastrointestinal involvement in TRAPS is common but can present diagnostic challenges. Following colon resection, histological examination revealed amyloid deposition, underscoring the importance of a comprehensive evaluation of these patients. Isolated colic amyloidosis has significant diagnostic and prognostic implications, warranting cautious monitoring and tailored management strategies. Treatment of TRAPS typically involves anti-inflammatory agents such as IL-1 inhibitors, with our patient experiencing clinical improvement on anakinra and canakinumab. Conclusion. This case report emphasizes the diverse manifestations of TRAPS and the importance of recognizing gastrointestinal complications, particularly isolated colic amyloidosis. Comprehensive evaluation, including histological examination, is crucial for identifying atypical disease presentations and guiding management decisions. Continued research is needed to elucidate the underlying mechanisms and optimize treatment strategies for TRAPS and its associated complications.

## 1. Introduction

Tumor Necrosis Factor Receptor-Associated Periodic Syndrome (TRAPS) represents an autosomal dominant hereditary autoinflammatory disorder [1] due to mutations in the TNFRSF1A gene, which encodes the tumor necrosis factor receptor (TNFR)-1 [2]. These mutations, found solely within the extracellular section of TNFR1, alter the receptor’s structure and ability to bind with the TNF ligand, boosting inflammatory responses by continuously activating various immune pathways. Secretion of proinflammatory cytokines, including IL-1β, culminates in unregulated inflammatory reactions [3]. Nonetheless, the precise mechanism linking the TNFRSF1A mutation to augmented IL-1β release is subject to ongoing investigation. Other potential contributors to the disease’s development include impaired receptor shedding, TNF-induced cell death, the generation of reactive oxygen species, and compromised autophagy. Patients exhibit an exaggerated response of their white blood cells to stimuli, resulting in heightened levels of proinflammatory cytokines [4]. Clinically, TRAPS is characterized by recurrent and protracted episodes of fever, abdominal and chest pain, arthralgia, myalgia, periorbital edema, conjunctivitis, and erythematous migratory skin rash [5,6]. Patients show elevated acute phase reactants and high serum amyloid A (SAA) levels during febrile episodes. Systemic autoimmune (AA) amyloidosis [7,8] stands out as a significant contributor to morbidity and mortality [8,9] within the TRAPS patient population. Biological agents, particularly interleukin (IL)-1 inhibitors, have proven substantial efficacy in preventing AA amyloidosis.

## 2. Case Presentation

We present the case of a 29-year-old woman with a history of recurrent episodes of abdominal pain, constipation, fever, lymphadenopathy, myalgia, and joint pain dating back to childhood. Despite extensive consultations with healthcare providers and specialists, a conclusive diagnosis was not performed until the age of 27, when, due to the persistence of febrile episodes, abdominal pain, arthralgias, and a persistent increase of inflammatory markers and SAA, genetic testing for autoinflammatory diseases was performed. The test revealed a heterozygous R92Q mutation in the TNFRSF1A gene [10,11,12], classified as a variant of uncertain significance (VUS). A non-confirmatory TNFRSF1A genotype, associated with episodes lasting ≥ seven days and myalgia symptoms, allowed for the diagnosis of TRAPS according to the 2019 EUROFER/PRINTO criteria. Noteworthy additional findings include dolichocolon, transverse colon ptosis, gastroesophageal reflux disease, hiatal hernia, familial hypercholesterolemia, arterial hypertension, allergic rhinitis, allergic bronchial asthma, arachnoid cyst, migraine, breast nodule, and vitamin D deficiency.

Subsequent genetic examinations unveiled H1299R (FVR2) heterozygous mutations in the Factor V gene [13], C677T (MTHFR 677) in the MTHFR gene [14], and 4G/5G in the PAI-1 gene [15].

Since the diagnosis of TRAPS, the patient has started anti-IL-1 therapy with anakinra 100 mg daily, with marked improvement in systemic symptoms and normalization of inflammatory markers. After seven months of treatment, due to difficulty in performing daily subcutaneous administration, the patient switched to canakinumab 150 mg every four weeks, with an initial good clinical response of systemic symptoms but persistence of gastrointestinal discomfort. In addition, she was administered budesonide, linaclotide, and mesalazine to treat gastrointestinal symptoms; inhaled corticosteroids and antihistamines for the allergic disease; and calcium channel blockers and beta-blockers for arterial hypertension. The patient has encountered complications and medication adjustments due to adverse effects, including reflex tachycardia and angina pectoris. Managing hypertension has proven challenging, necessitating alterations in medication. Exacerbations of asthma, migraines, and tachycardia episodes have prompted modifications in treatment plans and hospitalizations. In response to persistent and worsening tachycardia episodes, a loop recorder was surgically implanted for cardiac activity monitoring.

Despite the patient’s symptoms, MR enterography could not reveal significant findings two years before surgery. The MR examination did not exhibit signs of right colon amyloidosis, including diffuse wall thickening, intussusception, dilatation due to hypomotility, or luminal narrowing attributed to amyloid infiltration or ischemia.

**Figure 1:**
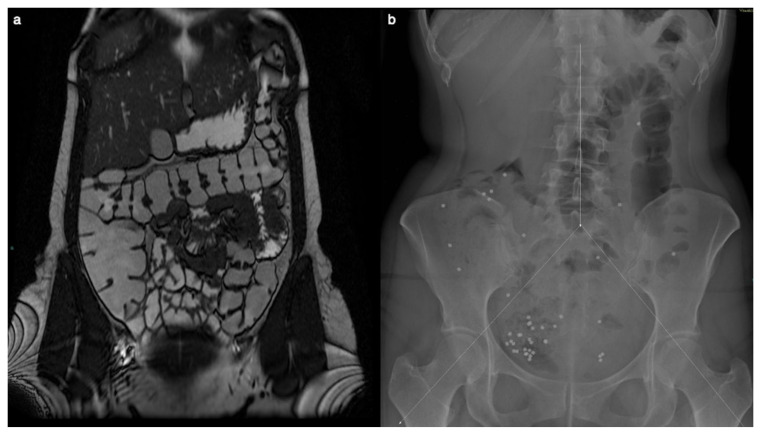
MR enterography showed no significant alteration two years before surgery, when the patient was already symptomatic. The Coronal T2-weighted image does not show signs of right colon amyloidosis (**a**). The plain abdominal X-ray is divided into three segments, and radiopaque markers are counted for each segment (**b**). Measurement of colonic transit time based on radio-opaque markers shows delayed colonic transit time (39/60 markers). The distribution of the markers, with a majority in the right region (31/39), suggests reduced motility at the level of the cecum and ascending colon.

Transit time and gastrointestinal motility exams were performed. The examination indicated a delayed colonic transit time, with only 39 of 60 markers progressing within the anticipated timeframe. Furthermore, the distribution pattern of these markers pinpointed areas of diminished motility, with the majority of those accumulating in the right region of the colon. This concentration suggested a potential impairment in the normal functioning of the cecum and ascending colon. Such detailed observations from the X-ray findings enhanced the understanding of gastrointestinal motility disorders and guided subsequent diagnostic and therapeutic decisions (Figure 1).

A colonscopic examination was performed without revealing any significant alterations; endoscopic biopsies were taken, leading to microscopic, unremarkable findings.

**Figure 2:**
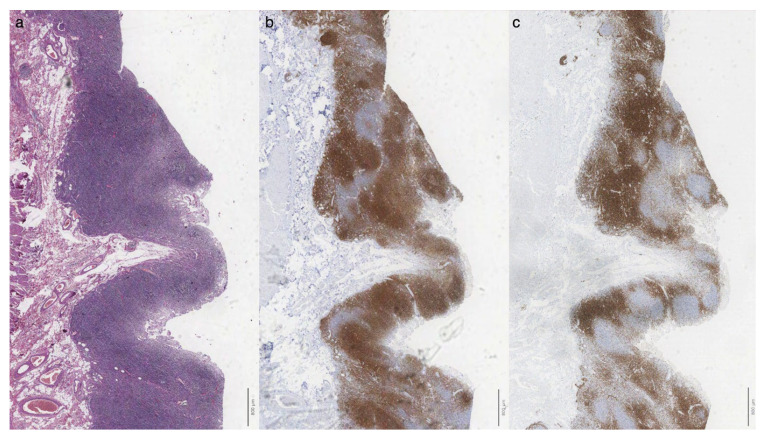
Dense infiltrate was observed within the lamina propria ((**a**), H&E stain) composed of small CD20+ B lymphocytes organized in follicles (**b**) admixed with a CD3+ T cell infiltrate (**c**). Scalebar = 800 microns.

Surgery was eventually pursued because of overwhelming intestinal obstruction involving the right colon and distal ileal resection. On histological examination, the walls of both the small and large intestines exhibited mucosal atrophy, particularly notable in the cecal region, accompanied by a dense lymphocytic infiltrate (Figure 2) within the lamina propria composed of a mixture of small-sized T cells (CD3+, CD5+) and small B-lymphocytes (CD20+, CD79a+, CD5-, BCL2+, CD10-, BCL6-), the latter organized in follicles with occasional germinal centers (CD10+, BCL6+, BCL2-).

**Figure 3:**
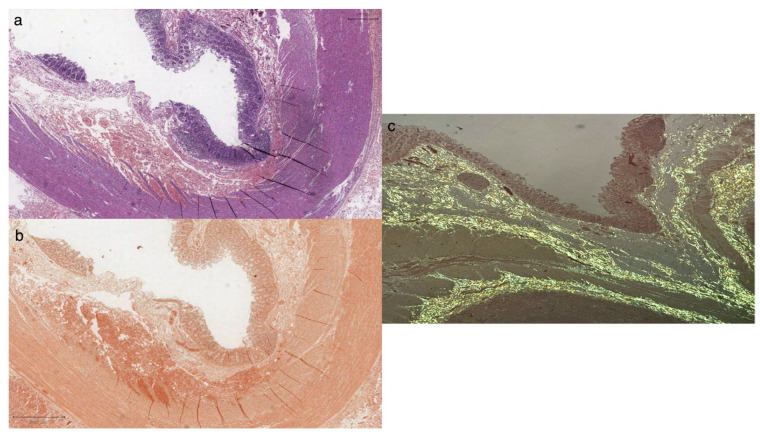
Histologic examination (**a**) revealed large intestine mucosal atrophy harboring amorphous material in the submucosa and muscularis propria (H&E stain), staining intensely for Congo Red along submucosal and muscular layers (**b**); observation under a polarized microscope showed apple-green (**c**) birefringence suggestive of amyloid deposits. Scalebar = 800 microns.

Scattered polytypic plasma cells (CD138+), rare CD30+/EBER-activated blasts, and occasional CD117+ mast cells were also observed. Histological staining with Congo Red revealed widespread amyloid deposits with intense apple-green birefringence on polarized observation (Figure 3). No signs of ganglionic hyperplasia were seen.

## 3. Results

TRAPS is a rare autoinflammatory disorder characterized by recurrent fever episodes and systemic manifestations such as abdominal or chest discomfort, joint pain, and skin rash. It is primarily linked to mutations in the TNFRSF1A gene, which encodes the TNFR1 receptor, and is inherited in an autosomal dominant pattern. The pathophysiology of TRAPS involves altered structure and function of TNFR1 due to mutations, leading to dysregulated inflammatory responses [16,17]. Despite extensive research, the precise mechanisms underlying TRAPS pathogenesis remain incompletely understood. However, mechanisms such as endoplasmic reticulum stress, impaired receptor shedding, TNF-induced cell death, oxidative stress, and compromised autophagy have been implicated [5,18,19].

TRAPS treatment typically involves anti-inflammatory therapies to mitigate symptoms and prevent disease flares. In this case, the patient received treatment with anakinra, canakinumab, and medications such as budesonide and mesalazine targeting gastrointestinal symptoms [20]. However, the patient experienced complications and medication adjustments, including reflex tachycardia and angina pectoris, highlighting the challenges of managing TRAPS and its associated comorbidities.

In the present case, genetic testing revealed a heterozygous R92Q mutation in the TNFRSF1A gene, confirming the diagnosis of TRAPS syndrome. This finding shed light on the patient’s longstanding history of recurrent abdominal pain, fever, and joint pain, which a definitive diagnosis could not have recognized. Moreover, additional, and potentially confounding genetic mutations were identified, including heterozygous mutations of H1299R (FVR2) in the Factor V gene, C677T (MTHFR 677) in the MTHFR gene, and 4G/5G in the PAI-1 gene. While the significance of these mutations in context and the link to genetic susceptibility to TRAPS remain fully elucidated, they underscore the complexity of the patient’s clinical presentation. They may have implications for disease management and prognosis.

Gastrointestinal involvement is common in TRAPS, with manifestations ranging from abdominal pain to gastrointestinal motility disorders. The evaluation of gastrointestinal transit time revealed a delay in colonic transit, with a higher concentration of markers in the right colon, suggesting impaired/reduced motility in the cecum and ascending colon. Histologic examination of the biopsies taken during the endoscopic procedure did not reveal any significant changes or amyloid deposition. This may be attributed to the superficial nature of the mucosal biopsies, which were not deep enough to detect amyloid deposition and its erratic distribution. This finding guided subsequent diagnostic and therapeutic decisions, ultimately leading to surgery for overwhelming intestinal obstruction.

Amyloidosis is a well-recognized complication of TRAPS, typically involving systemic deposition in organs such as the kidneys, thyroid, myocardium, liver, and spleen. However, the isolated finding of colic amyloidosis [21,22], as observed in our patient, is a rare occurrence and has not been extensively documented in the literature but should be taken into account as pseudo-obstruction carries a particularly grave prognosis, often not responding to promotility agents [23]. This finding expands our understanding of the spectrum of amyloid deposition in TRAPS and underscores the importance of considering gastrointestinal involvement in the disease pathology. It is worth noting that earlier studies on isolated colonic amyloidosis have described deposits primarily localized within particular colon regions, notably the rectum and transverse colon, as the most frequently affected areas [24,25].

Identifying colic amyloidosis in our patient has several clinical implications, highlighting the importance of a comprehensive histological examination even in the absence of overt gastrointestinal symptoms. Given the final results, it could have been performed before surgery for overwhelming obstruction on biopsy specimens from the endoscopic examination. The presence of amyloid deposition in the colon may have diagnostic and prognostic significance, potentially influencing treatment decisions and long-term management strategies [5,9].

Additionally, the discovery of isolated colic amyloidosis underscores the importance of cautious monitoring of gastrointestinal issues in TRAPS patients. While colic amyloidosis presents its own clinical challenges and management strategies, systemic amyloidosis remains a significant concern for these individuals, with a grim overall prognosis and a shorter survival time.

## 4. Conclusions

Identifying isolated colic amyloidosis in our patient is a unique feature of this case report. While systemic amyloidosis is a known complication of TRAPS, the isolated deposition of amyloid in the colon is rare and not extensively documented in the literature. This finding expands our understanding of the diverse manifestations of TRAPS and underscores the importance of considering gastrointestinal involvement in the disease pathology. This case report highlights the need for the comprehensive evaluation and management of gastrointestinal complications in TRAPS, as well as the value of histological examination in identifying atypical disease presentations and the pathological base of the disease.

## Data Availability

The data presented in this study are available on request from the corresponding author. The data are not publicly available due to the privacy policies of the centers involved in the study.
